# Exploring nightly variability in sleep apnea and changes in severity classification with multi night sleep testing in a population based cohort of young children

**DOI:** 10.3389/frsle.2026.1903809

**Published:** 2026-08-19

**Authors:** Solveig Magnusdottir, Ingibjorg Ingolfsdottir, Hugi Hilmisson, Laufey Hrolfsdottir, Magnus Birkisson, Erla Hallgrimsdottir, Groa Johannesdottir, Hannes Petersen

**Affiliations:** 1 MyCardio LLC, Denver, CO, United States; 2 Akureyri Hospital, Pediatrics, Akureyri, Iceland; 3 University of Iceland, Reykjavík, Iceland; 4 Institution of Health Science Research, University of Akureyri and Akureyri Hospital, Akureyri, Iceland; 5 Akureyri Hospital, Ear Nose Throat, Akureyri, Iceland; 6 Aarhus University, Aarhus, Denmark

**Keywords:** apnea hypopnea index, children, diagnostic accuracy, night-to-night variability, sleep apnea

## Abstract

**Background:**

Obstructive sleep apnea (OSA) is typically evaluated with single-night sleep studies. In adults, night-to-night variability (NtNV) is well documented, but less so in children. The current study evaluated NtNV in apnea events for misclassification in disease severity in young children to investigate if multi-night sleep-recordings may be clinically relevant to improve diagnostic accuracy and affect treatment decisions.

**Methods:**

Parents/guardians of healthy 4–9-year-old children participating in a study to evaluate prevalence of OSA were asked to record the child's sleep for up to 5-nights. This analysis is from children who completed three consecutive night sleep recordings.

**Results:**

Average age of participants was 6.1 years (*n* = 200, 58.0%-girls/42.0%-boys), prevalence of overweight/obesity was 25% and moderate-severe-OSA (AHI_3%_ ≥ 5) based on single-night testing protocol was 29.0%. Using the highest AHI_3%_ value from the three recordings, the first-night correctly identified 57.0% of participants and adding a second night identified 86.0% of participants, with 14% of participants not identified until the third night of sleep testing.

Bland-Altman plots (BA_plots_) demonstrated non-statistically significant mean-differences and 95% upper and lower-limits (LOA_95%_) close in absolute terms night-one/night-two; −0.024 events/hour-of-sleep LOA_95%_ [−5.062, 5.013], 8.0% of observations outside the LOA_95%_ (*p* = 0.894) and night-two/night-three; −0.009 events/hour of sleep, LOA_95%_ [−5.227, 5.209] and 6.0% of observations outside LOA_95%_, (*p* = 0.9620).

The intra-class correlation coefficient (ICC) for AHI_3%_ for all 3 nights was 0.45 [0.37, 0.54], *p* < 0.001, for night-one/night-two (0.44; *p* < 0.001), and night-two/night-three (0.44; *p* < 0.001). Cohens Kappa (0.304–0.462) and Brennan-Prediger Kappa (0.403–0.490), respectively indicated low-to-moderate interrater agreement, with quadratic-weighted Cohen's Kappa for the four ordinal severity categories of 0.37 and 0.48 for the two night-pairs, demonstrating NtNV, which is consistent with the variability demonstrated by the BA_plots_.

**Conclusion:**

Observed NtNV in apnea events affected severity categorization, suggesting that multi-night sleep testing may have clinical value and improve accuracy of sleep apnea evaluation in young children.

**Clinical trial registration:**

URL: clinicaltrials.gov/study/NCT05479201, identifier NCT0547920 .

## Introduction

Obstructive sleep apnea (OSA) is the most severe form of sleep disordered breathing (SDB) in children. OSA is characterized by repeated episodes of partial and/or complete upper airway obstruction causing intermittent hypoxia, hypercapnia, arousals, and sleep fragmentation that may affect the total sleep time (TST) and alter the sleep architecture with decrease in slow waves and sleep spindles that may affect the sleep recovery processes (Urbain et al., 2013; Punjabi et al.,  2020). Moderate - severe OSA is diagnosed in children when the apnea-hypopnea-index using 3%-desaturation criteria (AHI_3%_) is ≥5.0/hour of sleep (Marcus et al., 2012). In preschoolers (2–5-years old children) the primary cause of upper-airway blocking often includes tonsillar and/or adenoid hypertrophy, as during this age tissue growth may be disproportionate to the growth of the bony parts of the nasopharyngeal space (Chuang et al., 2022). Other common causes of OSA in otherwise healthy children are midface- and mandibular-deficiency, malocclusions (Maeda et al., 2014) and obesity (Alonso-Álvarez et al., 2014; Bin-Hasan et al., 2018), as allergic rhinitis and asthma (Lunn and Craig, 2011) can contribute to worsening symptoms. In the past decade understanding has been established of how intermittent hypoxia, sleep fragmentation and systemic inflammation (Gozal et al., 2008; Wang et al., 2023) caused by untreated OSA can lead to long-term morbidities including neurocognitive impairments, behavioral and/or mental diseases in children (Pereira et al., 2024; Reynaud et al., 2018; Song et al., 2016) and can contribute to development of hypertension (Kang et al., 2017), obesity (Di Sessa et al., 2022) and decreased quality of life (Mitchell and Kelly, 2006; Rosen et al., 2002).

Attended in-laboratory Polysomnography (PSG) is the reference standard to evaluate sleep in children. PSG studies are complex, costly, access is limited and studies are rarely performed over multiple nights. This may be problematic owing to physiological night-to-night variability (NtNV) in AHI.

The NtNV in AHI is well documented in adults but less so in children (Punjabi et al.,  2020; Roeder et al., 2020). Previously published studies that have utilized PSG to evaluate NtNV have reported a significant and practical NtNV in AHI and demonstrated an additional clinical and practical value in sleep testing for a second night. Multiple reasons can cause NtNV including but not limited to the first night effect (FNE) that can cause more arousals with more time spent awake during the sleep period, leading to compromised total sleep time and sleep quality. The duration of the sleep period spent in supine position and duration of Rapid Eye Movement (REM) sleep have both been reported to be less during first night of PSG testing which can affect recorded OSA severity, as sleep apnea events occur more frequently during both REM sleep and in the supine position. Therefore, respiratory parameters such as the AHI can be underestimated in a sleep study that is conducted as a single night of testing (Qin et al., 2022; Scholle et al., 2003; Verhulst et al., 2006; Xiao et al., 2026). Additionally, conventional sleep diagnostic, such as PSG and home sleep apnea testing (HSAT) measuring nasal airflow and respiratory effort, have limitations characterized by operational complexity, reduced patient tolerability and elevated technical failure rates, even more so in children than adults. Consequently, novel sleep apnea diagnostic platforms have emerged to optimize usability and expand diagnostic capacity, including offering multi night testing (Chiang et al., 2026a). “*These novel sleep apnea diagnostic tools can be considered in children*” (Marcus et al., 2012) and recent reviews have introduced the applicability of methods alternative to PSG for sleep evaluation in children with home sleep apnea testing (HSAT) getting more accepted. Simpler sleep testing options which have been validated and cleared for use in children may thus be more realistic as the first choice to evaluate OSA and the NtNV in AHI and may hold potential for improving access to sleep testing and accuracy, as well as being a management tool of treatment diagnostic intervention and outcomes (Chiang et al., 2026a; Garde et al., 2022; Landry et al., 2024; MacLean et al., 2026; Tan et al., 2015).

One such method is the SleepImage^®^ System a U.S. Food and Drug Administration (FDA) cleared and CE-certified in compliance with European Union Medical Device Regulation (EU-MDR) that is based on cardiopulmonary coupling (CPC) analysis reflecting integrated output of brainstem cardiorespiratory interactions modulated by electrocortical brain activity to measure sleep (Al Ashry et al., 2021b; Thomas et al., 2005, 2014). When the CPC-output is combined with oxygenation information (SpO_2_), a PSG-equivalent AHI is generated. The accuracy for diagnosis of the AHI_3%_ to categorize OSA has been validated in children and when compared to AHI_3%_ from PSG, it presents high sensitivity (0.88–0.95) and high specificity (0.84–0.97), to rule in and rule out sleep apnea at all severity levels (mild, moderate, and severe; Hilmisson et al., 2020).

Relatively few studies have evaluated NtNV in AHI in children. Studies that have utilized PSG included a wide age-range of children (Katz et al., 2002; Scholle et al., 2003; Verhulst et al., 2006) and studies that utilized HSAT in children also included a wide age-range, children with comorbidities and non-consecutive nights of sleep data (Orntoft et al., 2020; Yilmaz Yegit et al., 2023). These studies may therefore provide limited visibility of age-dependent sleep apnea and NtNV in children. This analysis aims to provide more clarity on the clinical role of multi-night sleep testing when evaluating OSA in young children (4–9-years of age;) by investigating the NtNV in AHI_3%_ in a community based sample of children, how it may affect severity categorization of sleep apnea from night-to-night and if it has clinical relevance.

## Methods

### Study design

The data was collected in the children's homes, administered by a single center in a prospective population-based cross-sectional study. The primary aim of the study was to evaluate the prevalence of OSA in healthy 4–9-year-old children residing in a geographically defined area in Northern Europe reflecting the broader local community. After approval from the local research ethics committee (VSN-22-096) the study was registered (NCT05479201) and invitations to participate were sent out *via* email with permission from the Department of Education and Public Health to parents/guardians of children in the defined age group. Parents/guardians interested in enrolling their child to participate were invited to an introductory meeting to discuss the objectives of the study, the measurements and procedures included in the study, and how they would be carried out. If interested to have their child participate, parents/guardians signed a consent form. Neither children nor parents were financially compensated for their participation the study. The study started on July 27th, 2022, and ended on June 19th, 2023. For a child to be included in the study two sleep studies with good signal quality of duration of ≥4 h of continuous sleep were required to capture a sufficient data with a full cycle of both REM and non-REM sleep. OSA categorization was based on the night with a higher AHI_3%_ value. A detailed description of methodology and primary results of the study outcomes have been reported (Ingolfsdottir et al., 2026). To evaluate NtNV included in this analysis, data from children with three (3) consecutive nights of good quality sleep recordings of ≥4-h in total sleep time (TST) was required. Study methods and results are reported following the Strengthening the Reporting of Observational Studies in Epidemiology (STROBE) statement for cross-sectional studies (von Elm et al., 2007).

### Sleep apnea evaluation

Parents/guardians were asked to record their child's sleep for up to five (5) nights with the SleepImage home sleep test (HST) that complies with the EU Medical Regulation (MDR CE-mark) and is FDA cleared for diagnosis of OSA in children from 2-years of age. The SleepImage System input comprises a finger-worn photoplethysmography (PPG) recording device, the SleepImage Ring (SR), that collects plethysmography signal (PLETH), oxygen saturation (SpO_2_) and movement (actigraphy). The SR connects *via* Bluetooth to the SleepImage Mobile Application, a non-Medical Device Data System (MDDS) that stores the data temporarily during the sleep recording. At the end of recording, the data is transferred from the MDDS for automatic analysis in the secure cloud-based portal, the SleepImage System. The method has been described in detail in several prior publications; it is based on analyzing dynamics in signals controlled by the autonomic nervous system (ANS) during sleep, that is, changes in coherence between pulse rate variability (PRV) and tidal volume variability (TVV) derived from the PLETH-signal. Coherence and cross-spectral power are calculated to generate sleep states and stages (Al Ashry et al., 2021b; Lee, 2012; Thomas et al., 2014). When the CPC-output is combined with SpO_2_ data, a PSG-equivalent AHI is generated. The accuracy for the AHI generated to categorize OSA has been validated in children and when compared to AHI from PSG, presents high sensitivity (0.88–0.95) and specificity (0.84–0.97), depending on OSA severity (Collop et al., 2011; Hilmisson et al., 2020). The SleepImage System is cleared for automated calculation of sleep states, stages and the AHI (K182618) with the option to “*manually override the apnea scoring*” to comply with regulations from the American Academy of Sleep Medicine (AASM). The metric of main interest in this study was the AHI_3%_ that is calculated from respiratory-induced pulse wave modulation (PWA) and PPG signal intensity changes resulting from changes in thorax pressure affecting hemodynamics during apneas, combined with SpO_2_ and the sleep analysis, including autonomic arousal detection that is included in identification of hypopneas (Chiang et al., 2026b). This study relied on autoscoring for the AHI_3%_. All sleep reports were manually evaluated and overread/edited by a sleep specialist blind to all information about participants other than age and sex. The autogenerated sleep output was adjusted when needed for (1) study start/end time and (2) artifacts caused by movements which were removed, including respiratory events as appropriate.

### Outcome measures

The primary outcome measures were to evaluate NtNV in AHI_3%_ to assess if multi-night sleep recordings (three-nights) may be beneficial to increase accuracy of severity categorization of OSA in young children. Classification of OSA-severity was defined based on 3%-desaturation criteria (AHI_3%_): no- or mild-OSA (AHI_3%_ < 5.0), moderate-OSA (AHI_3%_ 5.0–9.9) and severe-OSA (AHI_3%_ ≥10).

### Data analysis and statistical methods

Demographic data was presented, separately for children who were included and excluded in this analysis as percentages presenting proportions and means with 95% confidence intervals [CI_95%_], to assess potential bias in the cohort. Mean differences were tested using paired *t*-tests or proportion tests, whichever was appropriate.

To investigate NtNV in sleep measures, inter-night reliability was assessed, with higher reliability indicating less variability based on the intraclass correlation coefficient (ICC). The ICC, a widely used metric for evaluating constancy of continuous clinical data, was calculated using a two-way random-effects model for absolute agreement for individual measures. This model treats the specific nights as a random sample of possible nights, allowing generalization beyond those recorded. ICC value ≥0.70 was established as the minimum acceptable threshold for night-to-night reliability (Liljequist et al., 2014). Analysis was conducted for two specific sequences: from baseline to the second night and from second to third night. The variability in the primary outcome, AHI_3%_, was graphically analyzed using scatter plots, showing the actual values and Bland-Altman (BA) Plots, plotting the differences and means of paired observations, with the mean difference and 95% limits of agreement (LOA_95%_).

Interrater agreement was assessed by calculating Cohen's Kappa and Brennan and Prediger Kappa (PABAK). Because the four OSA-severity categories are ordinal, agreement across the full severity classification was additionally quantified using quadratic- and linear-weighted Cohen's Kappa, with 95% confidence intervals obtained by bootstrap resampling (5,000 replicates). The intra-class coefficient was calculated using a two-way random-effects model for absolute agreement. Finally, using Receiver Operating Characteristics (ROC), the data was evaluated to assess the diagnostic/classification changes of one night on the subsequent night(s), with the main metric of interest being the positive and negative predictive values. Python 3.10.17 was used for the analysis, including the numpy (1.26.4), pandas (2.3.3), scipy (1.15.3) and pingouin (0.6.1) with matplotlib (3.10.3) to generate graphics.

## Results

### Demographics of participants

The flow of participants is presented in Supplementary File 1. Children who only completed two nights of sleep recordings were excluded (*n* = 171) and only children with three consecutive nights of continuous sleep recordings with ≥4-h of TST were included in the analysis (*n* = 200). Comparison of characteristics of the children included and excluded are presented in Table 1, demonstrating no significant difference other than higher number of boys and children with reported snoring but not apneas in the excluded group. The average age of the children included was 6.1-years, 58.0% are girls and 42.0% boys, 29% with moderate-severe OSA on night-1 and 25.0% of the children were overweight or obese.

**Table 1:** Baseline characteristics of the children included (*n* = 200) and excluded (*n* = 171) from the analysis.

Characteristics	Included^a^	Excluded^a^	Included vs. Excluded^b^
Age (years: months)	6.1 [5.9, 6.3]	5.9 [5.6, 6.1]	0.136
Girls (%)	58.0 [51.2, 64.8]	43.2 [36.1, 50.4]	0.004
Caucasian (%)	104.0 [0.0,100.0]	100.0 [97.4, 100.0]	0.052
Asthma (%)	8.5 [4.6, 12.4]	8.6 [4.6, 12.7]	0.683
Allergy (%)	20.0 [14.5, 25.5]	8.6 [4.6, 12.7]	0.066
Gastro Esophageal Reflux (%)	8.5 [4.6, 12.4]	12.4 [7.7, 17.2]	0.206
Nocturnal Enuresis (%)	25.0 [19.0, 31.0]	26.5 [20.1, 32.8]	0.739
Positive score; Attention Hyperactivity DSM-V (%)	28.0 [21.8, 32.4]	29.6 [23.0, 36.1]	0.733
Overweight (BMI z-score ≥2.0) (%)	14.0 [9.2, 18.8]	15.1 [9.9, 20.2]	0.769
Obese (BMI z-score ≥2.5) (%)	11.0 [6.7, 15.3]	11.8 [7.2, 16.5]	0.798
BMI (kg/M^2^)	16.9 [16.6, 17.3]	17.1 [16.7, 17.6]	0.463
BMI z-score	0.82 [0.64, 1.00]	0.91 [0.72, 1.01]	0.474
Girls	0.82 [0.59, 1.05]	0.80 [0.53, 1.07]	0.911
Boys	0.82 [0.54, 1.09]	0.99 [0.74, 1.24]	0.352
Sleep Characteristics^*c*^			
Excessive daytime sleepiness (%)	24.5 [18.5, 30.5]	31.8 [24.9, 38.7]	0.117
Observed snoring (%)	26.0 [19.9, 32.1]	39.5 [32.2, 46.8]	0.005
Observed apnea (%)	9.5 [5.4, 13.6]	10.4 [5.9, 15.0]	0.771
Apnea Hypopnea Index_3%_ (AHI_3%_)-Night 1	4.1 [3.95, 4.34]	4.2 [3.92, 4.43]	0.834

Included in the analysis are children with three consecutive nights of sleep data of more than 4-hours in total sleep time.

^a^Descriptive statistics were presented as means with confidence intervals (CI_95%_).

^b^Difference comparing children included and excluded from the analysis (p- value).

^c^Derived from the Pediatric Sleep Questionnaire (PSQ) answered by parent(s).

### Nightly variability of sleep measures

The intra-class correlation coefficient (ICC) of sleep measures across the consecutive nights (night_1 to night_2 and night_2 to night_3) is reported in Table 2. The ICC for the AHI_3%_ comparing night_1 to night_2 [ICC = 0.44 (0.32, 0.54), *p* < 0.001] and night_2 to night_3 [ICC = 0.44 (0.32, 0.55), *p* < 0.001] suggests poor-to-moderate reliability. The ICC for AHI_3%_ for all 3 nights was 0.45 (0.37, 0.54), *p* < 0.001. Agreement across the four ordinal severity categories was consistent with these ICC values: quadratic-weighted Cohen's Kappa was 0.37 (CI_95%_ 0.25–0.48) for night_1 and night_2 and 0.48 (CI_95%_ 0.37–0.58) for night_2 and night_3 (linear-weighted 0.25 and 0.36, respectively) indicating low-to-moderate agreement.

**Table 2:** Intraclass correlation coefficients (ICC) of sleep measures for three consecutive nights^a^.

Sleep and respiratory measures	Night_1 to night_2^b^	*p*-value	Night_2 to night_3^b^	*p*-value
Sleep quality index (SQI)	0.72 (0.64–0.78)	< 0.001	0.75 (0.68–0.80)	< 0.001
Stable sleep (%)	0.70 (0.62–0.77)	< 0.001	0.71 (0.64–0.77)	< 0.001
Unstable sleep (%)	0.70 (0.62–0.76)	< 0.001	0.72 (0.64–0.78)	< 0.001
REM sleep (%)	0.40 (0.27–0.51)	< 0.001	0.39 (0.26–0.50)	< 0.001
Sleep efficiency (%)	0.44 (0.32–0.54)	< 0.001	0.43 (0.32–0.54)	< 0.001
Wake after sleep onset	0.32 (0.19–0.44)	< 0.001	0.39 (0.27–0.50)	< 0.001
Sleep fragmentation (%)	0.60 (0.50–0.68)	< 0.001	0.59 (0.49–0.67)	< 0.001
Apnea-hypopnea index_3%_	0.44 (0.32–0.54)	< 0.001	0.44 (0.32–0.55)	< 0.001
Hypoxic Burden (HB)	0.26 (0.12–0.38)	0.001	0.27 (0.13–0.39)	< 0.001
Respiratory Disturbance Index_3%_	0.71 (0.63–0.77)	< 0.001	0.71 (0.64–0.77)	< 0.001
Minimum heart rate	0.63 (0.55–0.71)	< 0.001	0.63 (0.54–0.71)	< 0.001
Maximum heart rate	0.54 (0.44–0.63)	< 0.001	0.57 (0.47–0.66)	< 0.001
Average heart rate	0.80 (0.74–0.84)	< 0.001	0.79 (0.73–0.83)	< 0.001

^a^Descriptive statistics were presented as means with confidence intervals (CI95%).

^b^ICC's of 0.70 or higher, are considered acceptable for night-to-night reliability.

Descriptive statistics for each sleep measure on each of the three nights are provided in Table 3. At the group level, no systematic NtNV were observed for any measure, including REM sleep (Nigtht_1 13.6%, Night_2 13.4% and Night_3 13.7%; *p* = 0.38) and AHI_3%_ (Night_1 4.1, Night_2 4.1 and Night_3 4.2 events/h; *p* = 0.37). Despite this stability of group means, within-child changes in REM sleep between consecutive nights were positively correlated with changes in AHI_3%_ (Pearson *r* = 0.42, *p* < 0.001), indicating that night with proportionally more REM sleep tended to show a higher AHI_3%_.

**Table 3:** Per-night sleep measures for children included in the analysis (*n* = 200) based on requirement of three consecutive nights of sleep recordings.

Sleep and Respiratory Measures	Night_1^a^	Night_1 [IQR]^b^	Night_2^a^	Night_2 [IQR]^b^	Night_3^a^	Night_3 [IQR]^b^	*P* value^c^
Sleep quality index (SQI)	70.5 ± 13.9	73.0 [62.0, 82.0]	70.1 ± 15.3	72.0 [59.0, 82.0]	69.5 ± 14.9	70.5 [59.0, 82.0]	0.687
Stable sleep (%)	62.3 ± 15.7	64.0 [52.0, 75.0]	61.5 ± 18.0	65.0 [48.0, 75.0]	61.1 ± 17.5	62.0 [49.0, 75.0]	0.935
Unstable sleep (%)	24.2 ± 13.2	21.5 [13.7, 33.0]	25.1 ± 15.3	21.0 [14.0, 35.2]	25.2 ± 14.8	23.0 [14.0, 34.0]	0.805
REM sleep (%)	13.6 ± 4.4	13.0 [10.0, 16.2]	13.4 ± 5.1	13.0 [10.0, 16.0]	13.7 ± 4.6	14.0 [11.0, 17.0]	0.380
REM sleep duration (min)	60.8 ± 22.7	57.6 [44.8, 74.7]	59.2 ± 23.3	57.6 [42.7, 74.7]	61.1 ± 21.8	59.7 [44.8, 74.7]	0.379
Sleep efficiency (%)	86.5 ± 5.4	86.0 [83.0, 91.0]	86.8 ± 5.6	87.0 [83.7, 91.0]	86.1 ± 5.9	86.5 [83.0, 90.0]	0.235
Wake after sleep onset (min)	70.2 ± 29.9	68.3 [49.1, 90.1]	68.3 ± 32.7	64.0 [44.8, 85.3]	72.5 ± 33.2	68.3 [46.9, 91.7]	0.186
Sleep fragmentation (%)	5.6 ± 5.4	4.0 [2.0, 7.0]	5.8 ± 6.3	4.0 [2.0, 8.0]	5.9 ± 5.3	4.5 [2.0, 8.0]	0.185
Apnea-hypopnea index_3%_	4.1 ± 2.2	3.8 [2.5, 5.4]	4.1 ± 2.6	3.5 [2.3, 5.2]	4.2 ± 2.5	3.6 [2.3, 5.4]	0.373
Respiratory disturbance index_3%_	15.0 ± 5.5	14.5 [10.9, 18.4]	14.8 ± 5.6	13.9 [10.6, 18.1]	15.3 ± 6.3	14.1 [10.7, 18.6]	0.171
Hypoxic Burden (HB)	4.9 ± 3.3	4.2 [2.9, 6.3]	5.1 ± 3.5	4.0 [2.9, 6.4]	4.8 ± 2.9	4.2 [2.9, 6.2]	0.803
Minimum heart rate	50.8 ± 5.1	51.0 [47.0, 54.0]	50.8 ± 5.0	51.0 [47.0, 54.0]	50.6 ± 5.0	51.0 [47.0, 54.0]	0.245
Maximum heart rate	123.4 ± 10.3	123.0 [117.0, 130.0]	122.6 ± 10.2	122.0 [114.7, 129.0]	122.5 ± 9.9	123.0 [116.7, 127.0]	0.248
Mean heart rate	78.5 ± 8.3	78.0 [73.0, 84.0]	78.5 ± 7.8	79.0 [73.0, 84.0]	78.3 ± 8.3	78.0 [73.0, 84.0]	0.316

^a^Descriptive statistics presented as means with ± standard deviation (SD).

^b^Interquartile range (IQR).

^c^Friedman, N1 = N2 = N3.

### Classification and re-classification of OSA category

**Figure 1:**
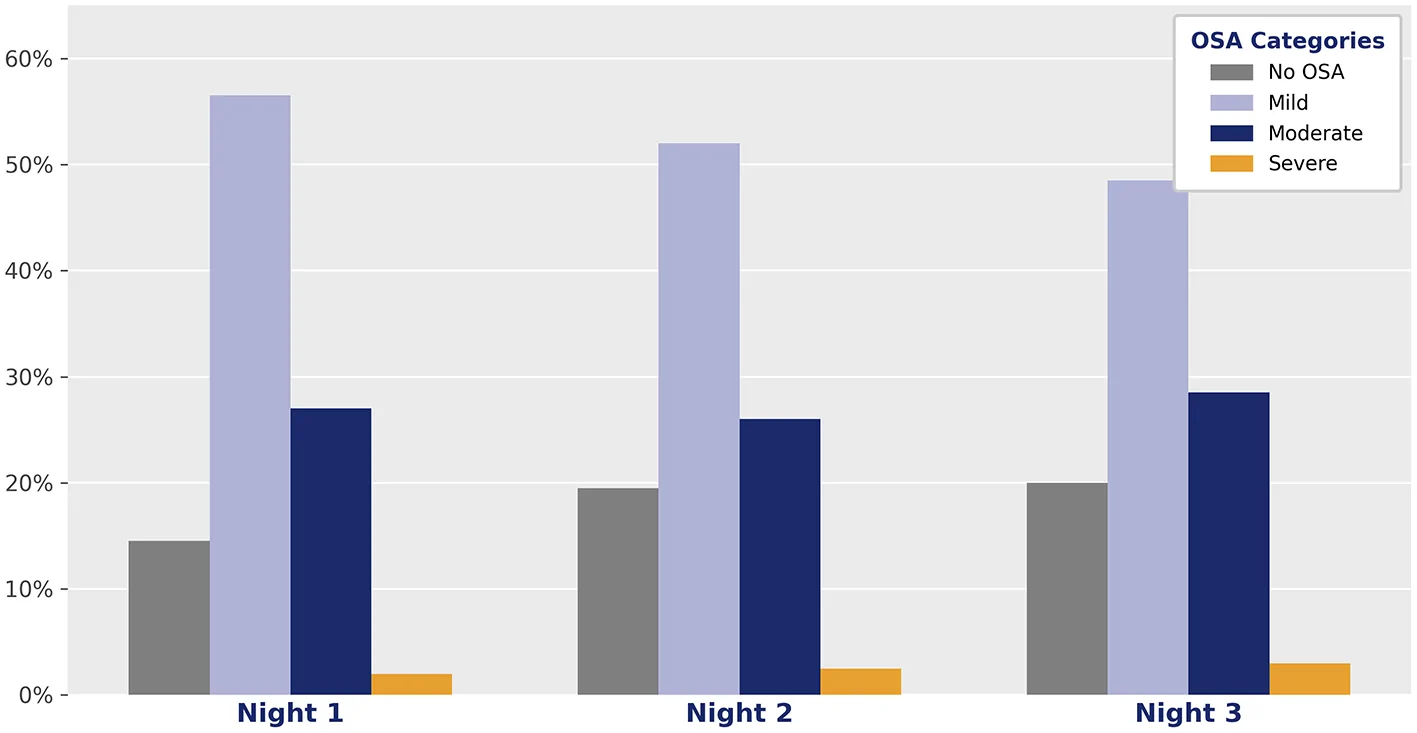
Demonstrates changes in OSA categories with adding a second and third night of sleep testing (*n* = 200).

The night-to-night variability (NtNV) in AHI_3%_ for classifications of OSA-category was evaluated on night_1 vs. night_2 and night_2 vs. night_3. Comparing night_1 and night_2, 101 children (50.5%) changed OSA severity category and 94 (47.0%) changed categories between night_2 and night_3 (Figure 1, Tables 4A, B and 5). These counts reflect the individual children who moved between categories (the off-diagonal cells of Tables 4A, B); smaller net changes in the number of children per category (Tables 4A, B) understate this individual-level variability. Using a binary threshold, 57 children (28.5%) and 54 (27.0%) advanced into the moderate-severe category comparing results on night_1 and night_2 and night_2 and night_3, respectively. Receiver Operating Characteristics (ROC) analysis was performed to investigate possible classification changes with an additional night(s) of sleep recordings (second- and third night). First, night_1 was compared to night_2. Then, combining the result of night_1 and night_2 where a higher AHI_3%_ on either night was utilized for OSA-categorization and compared to results from night_3 (Table 6). On night_1, 58 (29%) of the children were classified as positive for moderate-severe OSA, and 57 (28.5%) were classified positive on night_2, resulting in low sensitivity when night_2 is treated as the reference. When combining night_1 and night_2 into a single classification based on the night with higher AHI_3%_, 86 children (43%) were classified as positive for moderate-severe OSA. On night_3, 14 children were additionally identified as positive who were previously negative for moderate-severe OSA.

**Table 4a:** Presence of OSA on the first compared to the second night and diagnostic agreement between the nights in the children with recordings on three consecutive nights (*n* = 200).

		Night_2			
		AHI_3%_<2	AHI_3%_ 2–5	AHI_3%_ 5–10	AHI_3%_ ≥10
Night_1	AHI_3%_ < 2	12	14	3	0
	AHI_3%_ 2–5	23	65	24	1
	AHI_3%_ 5–10	4	24	22	4
	AHI_3%_≥10	0	1	3	0

**Table 4b:** Presence of OSA on the second- compared to the third-night and diagnostic agreement between the nights in the children with recordings on three consecutive nights (*n* = 200).

		Night_3			
		AHI_3%_<2	AHI_3%_ 2–5	AHI_3%_ 5–10	AHI_3%_ ≥10
Night_2	AHI_3%_ < 2	20	15	4	0
	AHI_3%_ 2–5	19	59	25	1
	AHI_3%_ 5–10	1	21	26	4
	AHI_3%_≥10	0	2	2	1

**Table 5:** Changes in sAHI_3%_ (*n* = 200).

	Night_1 and Night_2			Night_2 and Night_3		
	Night_1	Night_2	Difference	Night_2	Night_3	Difference
sAHI_3%_ < 2	29	39	10	39	40	1
sAHI_3%_ 2–5	113	104	9	104	97	7
sAHI_3%_ 5–10	54	52	2	52	57	5
sAHI_3%_≥10	4	5	1	5	6	1

**Table 6:** Receiver operating characteristics (ROC) based on categorization of identifying moderate sleep apnea (sAHI ≥ 5.0) and accuracy utilizing two-night compared to three-night protocol.

Moderate-severe OSA (sAHI ≥5.0 events/hr)							
	Night_1 vs. Night_2				Combined Night_1/Night_2 vs. Night_3		
		Night_1				Night_1	
		Negative	Positive			Negative	Positive
Night_2	Negative	114	28	Night_2	Negative	100	14
	Positive	29	29		Positive	37	49
	Sensitivity	50.9%			Sensitivity	77.8%	
	Specificity	79.7%			Specificity	73.0%	
	Accuracy	71.5%			Accuracy	74.5%	
	LR+	2.509			LR+	2.880	
	LR-	0.616			LR-	0.304	
	PPV	0.500			PPV	0.570	
	NPV	0.803			NPV	0.877	
	Cohen's κ	0.304			Cohen's κ	0.462	
	PABAK	0.403			PABAK	0.490	

Test result parameters reported as decimal value. Sleep apnea category is based sAHI, SleepImage Apnea Hypopnea Index; Brennan and Prediger, PABAK; Cohen's κ, Cohen's kappa; Night-1, N1; Night-2, N2; Night-3, N3; LR+, positive likelihood ratio; LR-, negative likelihood ratio; PPV, positive predictive value; NPV, negative predictive value.

### Graphical analysis

**Figure 2:**
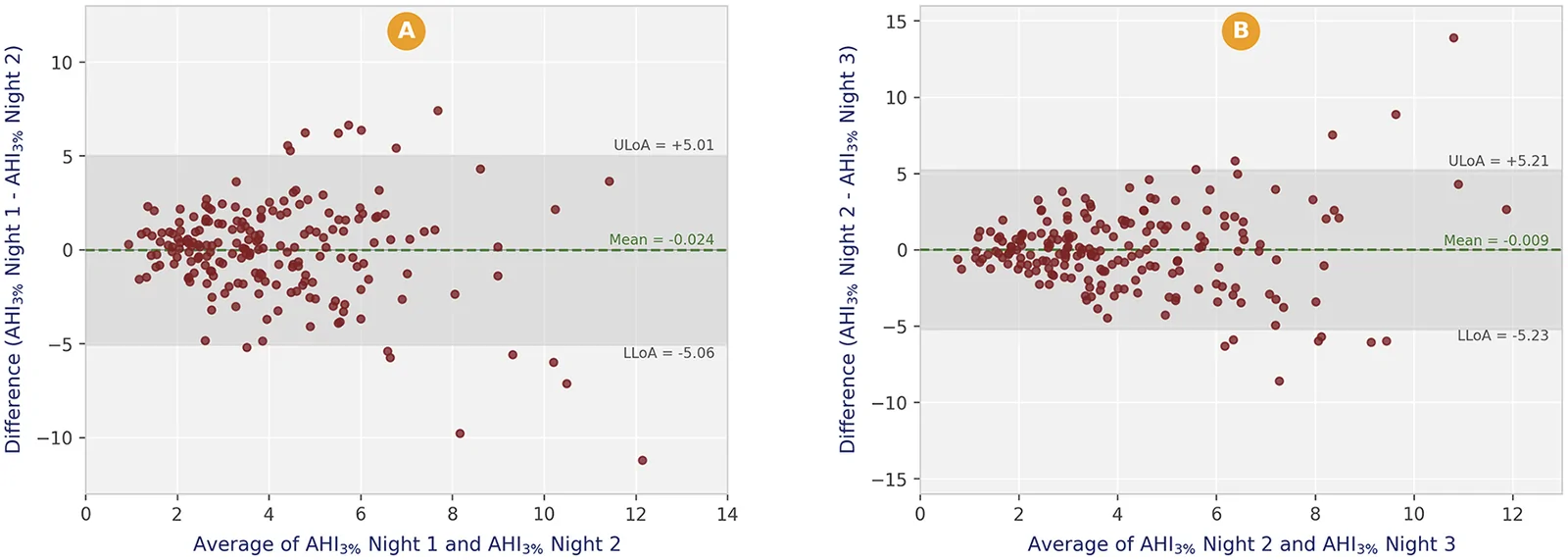
Bland-Altman plots demonstrating the apnea-hypopnea index (AHI_3+_) in the 200 children on: **(A)** First night and second night and **(B)** Second and third night. The dotted line represents mean of differences, while the grayed area marks the upper and lower limits of agreement respectively.

Bland-Altman plot of the AHI_3%_ are presented in Figure 2. Differences in AHI_3%_ were of both positive and negative values and tendency toward greater differences when averages increase at approximately a mean of 3-events/hours of sleep. This results in 95% limits of agreement of approximately +/−5-events/hours of sleep, without any clear upward or downwards bias, evidenced by non-statistically significant mean differences and the fact that the upper and lower limits are close in absolute terms. On night_1/night_2, a non-statistically significant mean of differences, −0.024 events/hours of sleep with LOA_95%_ [−5.062, 5.013] and 8.0% of observations outside the LOA_95%_ (*p* = 0.894). Results are similar when comparing night_2/night_3, with non-statistically significant mean difference, −0.009 events/hours of sleep, LOA_95%_ [−5.227, 5.209] and 6.0% of observations outside LOA_95%_, (*p* = 0.9620).

**Figure 3:**
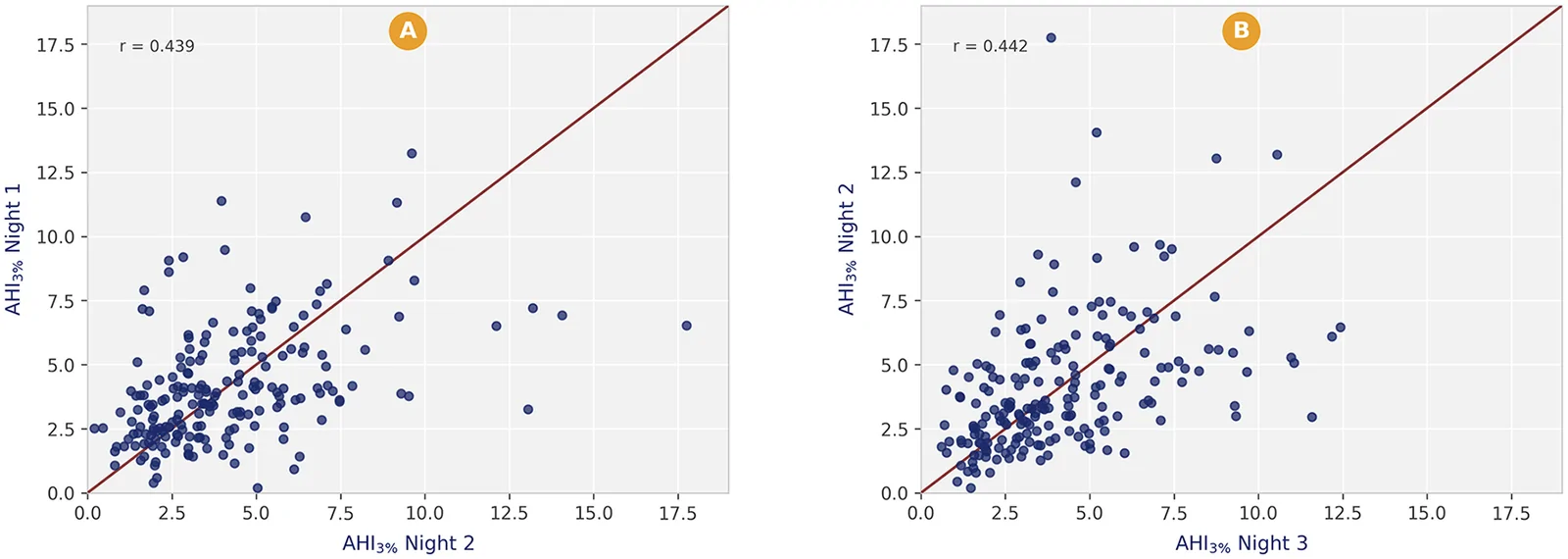
Scatter plot demonstrating measurements of the apnea-hypopnea index (AHI_3+_) recorded in the 200 children on: **(A)** First night and second night and **(B)** Second night and third night, and relation to line of identity.

Scatter plots are provided for reference to demonstrate the actual AHI_3%_ values (Figure 3). Points are located fairly close to the line of identity, especially regarding the lower values. Pearson correlation coefficient (*r* = ± 0.439 and *r* = ± 0.442) suggesting a notable, but not strong relationship. There was no pattern indicating that the measurements were systematically higher on the first/second or second/third night or the other way.

## Discussions

This analysis examined NtNV in AHI_3%_ and whether implementing multi-night sleep testing for sleep apnea evaluation may be beneficial to improve accuracy of severity categorization of OSA in young children. Findings suggest that single-night sleep assessment may not be adequate, and that subsequent night(s) testing may be beneficial based on NtNV in AHI_3%_ to optimize accuracy of OSA classification. As described above, the cumulative prevalence of moderate-severe OSA (AHI_3%_ ≥5) increased with additional nights of sleep testing, from 29.0% on a single night to 43.0% using the higher of the first two nights and 50.0% across all three nights; single-night prevalence was similar across nights (29.0% night_1, 28.5% night_2 and 31.5% night_3), indicating the increase reflecting NtNV rather than a systematic rise in severity. A poor-to-moderate reliability and a substantial variability in AHI_3%_ were observed possibly owing to physiological NtNV, indicating that multi-night testing may possibly be beneficial for ruling out moderate-severe OSA.

These findings should be interpreted at both; (1) Group level, were there was no systematic bias between nights. Bland-Altman mean differences were small and non-significant, and group mean values for AHI_3%_ and for sleep architecture, including REM sleep, did not differ significantly across the three nights. (2) Individual level, where agreement was however limited, as reflected by the wide Bland-Altman limits of agreement (approximately ±5 events/h), the low-to-moderate ICC and weighted Kappa, and the reclassification of approximately half of the children between consecutive nights. The absence of group-level bias therefore does not imply that a single night reliably classifies an individual child.

In the clinical setting when evaluating children suspected of suffering from OSA, re-test decisions need to be made in the presence of a negative test result on the first night. The results of this analysis that 86% of children with moderate-severe OSA were correctly identified with two-night testing protocol and 14% missed are similar to reports from PSG studies (Scholle et al., 2003; Verhulst et al., 2006) support a significant and practical NtNV in AHI_3%_ and that there is additional clinical value in sleep testing for a second night. The decreasing rate of additional positives with the third night of testing may suggest that the decision to test more than two nights needs to be weighed against the burden on the child and in light of the clinical suspicion of high pre-test probability. This is further supported by the ICC, Kappa values and positive predictive values, that were all low to moderate, demonstrating the variability in AHI_3%_, and variability in disease classification between nights. A three-night protocol for accurate sleep assessment, including AHI_3%_ has previously been suggested in a cohort of older individuals (Ding et al., 2025), and by an expert panel on multi-night testing for OSA in adults (Fricke et al., 2026) and may therefore plausibly also be appropriate for children.

Auto scoring of sleep studies can improve consistency by eliminating human judgement and interscorer variability, which in manual scoring of AHI from PSG studies is estimated in the range of 25%−45% (Thomas et al., 2020). Sleep evaluation based on changes in sympathetic and parasympathetic shifts rather than in EEG activity may add a measurement variability to sleep state, sleep staging and respiratory event scoring that contributes to measurement variability when compared to PSG. The CPC-method is based on coherence and coupling of changes in respiration and HRV that adds stability to the analysis. There are though multiple reasons where the two methods differ: 1) The CPC sleep analysis reports REM and NREM sleep, with the NREM category having two sleep stages (Stable- and Unstable sleep). This complicates a direct comparison of NREM-sleep stages to the three stages of NREM sleep in PSG, and as the CPC-method is based on scoring of 2.1-minute-long windows of data when PSG is based on 30 second windows (epochs). Regardless, a thigh temporal relationship has been observed between Stable sleep and slow-wave power (SWS; Thomas et al., 2014) when a closer relationship is observed with cyclic alternating pattern (CAP/non-CAP) scoring (Lee, 2012). (2) Though a high sensitivity, specificity and agreement have been reported from comparison of respiratory events (AHI_3%_) in both children and adults (Al Ashry et al., 2021a; Hilmisson et al., 2020) the CPC-method utilizes recorded ANS-arousals for hypopnea scoring when the PSG-method is based on cortical arousals (EEG). Not all ANS-arousals necessary translate into EEG-arousals, with the CPC-method possibly reporting higher number of AHI_3%_ than PSG. Therefore, rather than looking at strict comparison of sleep states, stages, arousals and respiratory events between the two methods, a higher value may be in utilizing how the two methods can complement each other in symptom evaluations as they may provide different clinical and functional information.

Furthermore, our results are similar to a study evaluating OSA with HSAT in 30 children and adolescents (7–18-years old), that correctly identified 83% of patients with OSA on night_1, finding a clinically relevant NtNV in AHI. The prevalence of OSA in this cohort was 80% when based on one night of sleep testing (Orntoft et al., 2020). A more recent PSG study evaluating test-retest reliability of AHI in children with moderate-severe OSA demonstrated high NtNV in AHI, cautioning that relying solely on a single-night PSG may cause misclassification and inaccurate diagnosis (Xiao et al., 2026). A recent systematic review looking at NtNV comes to a similar conclusion, that NtNV in AHI may cause children to be misdiagnosed by single-night PSG diagnostic sleep studies (Qin et al., 2022). As multi-night PSGs are not feasible, exploring NtNV using multi-night home-based portable sleep monitoring that has demonstrated substantially equivalent agreement levels with PSG from a single night may offer improved understanding in sleep measures and associated health consequences, both in clinic and research.

Patients may have more arousals with more time spent in wake, compromising total sleep time and sleep quality on the first night compared to consecutive nights. This phenomenon is known as the first night effect (FNE) that may cause NtNV in sleep and respiratory parameters represented when comparing consecutive nights. FNE has been estimated using in-laboratory PSG in cohorts of children of wider age range including sub-analysis of younger age-groups (Agnew et al., 1966): (1) Scholle et al. (2003) included 130 children and adolescents in their study and found that of the 37 participants aged 2–6-years, the 9 children diagnosed with OSA (prevalence 24%) had significantly higher number of arousals on the first-night compared to the second-night and 86% of those diagnosed with OSA based on two-night protocol were identified on the first night or false negative rate of 14% (Scholle et al., 2003). (2) Verhulst et al. (2006) evaluated 70 children with 22 children in the age-group of 2–6-years-old and found that the first night of PSG sleep study identified 86% of children in this age group correctly with OSA compared to the second night, or false negative rate of 14% (Verhulst et al., 2006). Other studies that have evaluated FNE and NtNV were based on data from recordings of non-consecutive nights (Katz et al., 2002) or included children with other comorbidities (Li et al., 2004). Studies evaluating FNE/NtNV in children generally agree that REM sleep is less on the first night with sleep efficiency improving on the second night (Li et al., 2004; Scholle et al., 2003; Verhulst et al., 2006). In children, obstructive apneas are known to occur more frequently during REM sleep and therefore respiratory parameters such as the AHI can be underestimated if REM-sleep duration is decreased. In our cohort, REM-sleep duration did not increase systematically from the first to later nights at the group level; instead, within child night-to-night changes in REM sleep were positively associated with changes in AHI_3%_ (*r* = 0.42, *p* < 0.001). In a linear mixed-effects model, the change in REM sleep accounted for approximately 20% of the variance in the night-to-night change in AHI_3%_ (marginal *R*^2^ = 0.20), indicating that fluctuation in REM sleep is one contributor rather than the principal driver of the NtNV in AHI_3%_. This association is consistent with a meta-analysis in adults in which between-night differences in REM sleep time were positively associated with differences in AHI (Roeder et al., 2020), and with the recognized predominance of obstructive respiratory events during REM sleep in children (Spruyt and Gozal, 2012). These findings suggest that individual fluctuations in REM sleep contribute to the observed variability in AHI_3%_, rather than a uniform first-night effect on REM-sleep duration.

Both physiological and technical factors contribute to NtNV in the AHI. In children, physiological variables include total sleep time, REM sleep time, body position, movements, and shifts in sleeping posture. Technologically, conventional in-laboratory PSG is limited by high manual inter-scorer variability—estimated between 25 and 45%—which particularly impacts the quantification of lighter sleep stages, arousals, and hypopneas (Punjabi et al.,  2020). In contrast, the SleepImage SaMD utilizes automated analysis for sleep states, stages and respiratory events, with an option for manual editing of respiratory events, thereby reducing human scoring variability. However, because its hypopnea scoring relies on autonomic arousals—which may not always correspond to electroencephalogram (EEG)-recorded cortical arousals—the system may yield higher AHI_3%_ values compared to traditional PSG (Chiang et al., 2026b).

Multi-night sleep recordings have the advantage of including aggregate information including NtNV assessment which provides both more extensive and more reliable data on the nightly physiological changes in sleep and sleep apnea to prevent possible missed diagnosis or to over-diagnose.

Adenotonsillectomy (AT) has long been recommended as first-line therapy in children with adenotonsillar hypertrophy and OSA (Marcus et al., 2012) which has made this procedure the most common ambulatory surgical procedure performed on children in the USA (Patel et al., 2014). Both the American Academy of Pediatrics (AAP) (Marcus et al., 2012) and AASM (Kirk et al., 2017) recommend objective sleep evaluation prior to surgery. Regardless of this recommendation, a survey among pediatric otolaryngologist in the USA found that fewer than 6% of responders referred children with OSA for a PSG study prior to surgery “most of the time” (Friedman et al., 2013). Using clinically validated home sleep testing devices that are cleared to have comparable output to PSG for children, with data from multiple nights in the child's own bed at home, has thus an additional benefit of offering holistic information from the child's “real-life” circumstances during baseline evaluation. Such testing procedure could prevent an unnecessary or premature surgical intervention as some children may benefit from watchful waiting with supportive care (WWSC) as reported by the childhood adenotonsillectomy study (CHAT) where over 40% of children had a spontaneous resolution of their disease when evaluated with one-night PSG at baseline and after period of 7-months (Marcus et al., 2013). Although AT has been reported to be effective in treating OSA in children without co-morbidities postoperative residual disease is prevalent and even more so in children with comorbidities, including children who suffer from overweight or obesity (Imanguli and Ulualp, 2016). Implementing treatment tracking in this population adds further value as some of these children will need additional intervention, possibly including positive airway pressure therapy (PAP) and/or orthodontic treatments. For children treated with PAP, monitoring therapy based on blood oxygenation and sleep quality offers an opportunity to improve clinical care and outcomes as has been reported in adults, where a significant discrepancy is often reported between the PAP download reports that may be useful to assess usage and leak, but may have the potential to underestimate residual apnea burden (Malhotra et al., 2026).

## In summary

The NtNV in apnea events observed in this study reporting poor-to-moderate night-to-night reliability in AHI_3%_ affected severity categorization of OSA. The study suggests there may be clinical value in implementing multi-night testing protocols for improved accuracy in severity categorization of obstructive sleep apnea in young children. Further studies are required to evaluate if multi-night testing improves diagnostic precision in the clinical decision-making process and improves outcomes.

## Strengths and limitations

Strengths of this study include a relatively large sample size in healthy children of a defined age group of preschoolers (4–6-years) and young school-aged (6–9-years) children. Sleep recordings were conducted over three consecutive nights with a clinically validated home sleep test cleared for use in children, allowing us to evaluate NtNV in sleep apnea and other sleep parameters. Participants were sleeping in their natural sleep environment at home, eliminating the effects of unfamiliar environment and major change in routines that may affect their sleep and test results. The simple form factor of the sleep-test being a ring-device that was not intimidating to the children and often excited them to sleep with their “Dream Ring”. This simplicity makes it easy for participants to apply the test in the same manner every night and therefore variability in application of the test is unlikely to affect their normal sleep habits. The data is automatically analyzed both for sleep staging and apnea calculations, eliminating the inter-scorer variability.

Limitations of the study are that all participants are from the same geographical area which may be viewed as both a strength and a limitation. All the children included in the analysis are of Caucasian origin, which is a limitation, and the findings may therefore not apply to children living in other geographical locations and/or children of other races or ethnicities. Children who did not have three-consecutive recordings of sleep were excluded from this analysis, causing higher number of girls to be included. It is unknown if that may have inadvertently caused selection bias. This was a voluntary study that all children in the predefined age group living in the study area were offered to participate, making it possible that parents /guardians of children with sleep difficulties may have been more interested in their child's sleep and health and therefore more likely to sign up for the study. This study was completed in a community sample and may thus not reflect the patient population that is seen in sleep clinics. The HST collects and reports movement, but it does not report body position as the device is worn on the finger and it does not record temperature. Body position can affect sleep apnea severity and temperature can affect sleep quality which possibly may compromise total sleep time during the sleep period with secondary effects on sleep apnea severity. PSG sleep studies were not part of the study; it is possible that use of HST may underestimate OSA severity. In contrast, using the highest AHI_3%_ may have caused overestimation of OSA severity.

## Data Availability

The data collected during this study is not publicly available due to General Data Protection Regulations (GDPR) reasons but may be provided under specific circumstances from corresponding author upon reasonable request.
