# Supplementary material for: Exploring nightly variability in sleep apnea and changes in severity classification with multi night sleep testing in a population based cohort of young children
Source: Front Sleep. 2026 Aug 19;5:1903809. doi: 10.3389/frsle.2026.1903809 (PMC13533644; doi:10.3389/frsle.2026.1903809)
Supplement: Supplementary file 1 [file Supplementary_file_1.docx]

Supplement 1. Flow of children with parental consent to participate in the study (n=386). Thirteen children did not finish answering included questionnaires and recording their sleep for two nights and were excluded. Children with chronic conditions (severe asthma, or other severe respiratory and pulmonary diseases, genetic disorders that may affect neurocognitive development and/or behavior, muscular dystrophy, and other neuromuscular disorders) were excluded from the final analysis (n=2). Children that did not have at least one full night of sleep recordings on school- and non-school nights were excluded from this analysis that includes sleep recordings from two hundred children.

Parents of 386 children signed a consent for their children to participate in the study.

13 children did not finish recording their sleep for the requested 2-nights.

373 children finished recording sleep for >4-hours over 2-nights requested for diagnosis of sleep apnea and returned all required questionnaires.

Children with chronic diseases were excluded from the analysis: a child with Down’s syndrome and a child with Noonan syndrome.

371 children were included in the final analysis to “*Evaluate prevalence of sleep apnea in children residing in the general population*”.

Recordings for 171 children did not meet the required quality criteria for this analysis of having three nights of sleep recordings with good signal quality with ≥ 4 hours of total sleep time.

Included in the analysis is data from 200 children.
